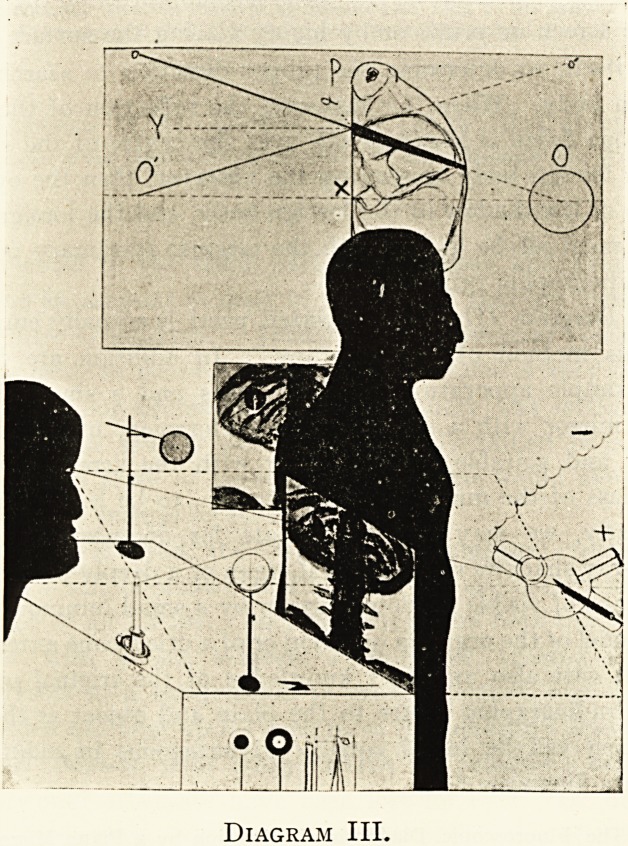# Design for an Elementary Radiographic Camera

**Published:** 1911-06

**Authors:** William Cotton


### DESIGN FOR AN ELEMENTARY RADIOGRAPHIC CAMERA.

BY

WILLIAM COTTON, M.D.

WHENEVER in systematic clinical work the radiographic centre of the X-ray focus tube is used throughout in some definite relation to the plane of the X-ray picture, then (as was suggested by Mr. Punch now some years ago) whether we know it or not we are employing a kind of X-ray *camera*. Just as in ordinary photography we employ a modification of the long-known "camera obscura," or may in copying drawings employ the "camera lucida" in some form, so in radiography we may quite rationally speak of using a sort of camera, which for want of a better name may be called the "Camera Aperta." The human eye itself is a camera, the comparison of which with other cameras just breaks off when it is becoming most interesting. The object of the present paper is to describe a somewhat more vertebrate type of camera for X-ray purposes than is usually to be found, and to suggest a few purposes for which it must be found highly advantageous or even essential.

The principle involved is that of keeping the radiographic centre always opposite the middle point or centre—that is, the point of intersection of the two diagonals—of the plate or screen at the time of exposure, while at the same time the radiographic centre is kept free to move to and from the plane of delineation. I first made this suggestion over two years ago.[1] Up to then, as far as I know, the prevailing practice was that of an ordinary photographer, using his swing back or his rising front indiscriminately and at haphazard.

It is curious how far the analogy and homology between ordinary and X-ray photography can be carried. Thus the radiographic centre corresponds with the optical centre of the combination of lenses used, or even more nearly to the pinhole of the pinhole camera. The fluorescent screen is both the ground glass focussing screen and the view finder. The plumb-line often used is analogous to the level. The light tight bag is the dark slide. Both the X-ray and photographic negatives present us, on development, with blackened high lights. The iris diaphragm is used habitually in both arts. Not less curious are some of the differences. In X-ray photography the object is between the plane of delineation and the radiant source, the image on the fluorescent screen and on the X-ray print is erect and reversed, while in the case of the ground glass screen of the photographer's camera the image is direct and inverted; and it is only now beginning to be dimly surmised by the majority of workers that in the matter of form and perspective the X-ray negative alone truly corresponds point for point with the ordinary photographic print or drawing taken from the original station point (in reference to the object and to picture plane) of the radiographic centre itself.

However this may be, in Diagram I I have tried to show the essential parts of a good practical workaday radiographic camera. As regards materials, they must of course be suitably strong, and electrically non-conducting where necessary.

The apparatus consists of *Baseboard*, of *Upright*, and of one or more *Brackets*.

[1] "The Principle of Proportional Representation in Clinical Radiography," *Practitioner*, 1909, lxxxii. 413.

The *Baseboard*, whose centre is marked X, has drawn on it rectangular outlines of half, of whole and of double-plate size, on which to place these plates in their customary light-tight wrappings, $x_{\prime}$ and $x_{\prime\prime}$ are subsidiary centres laterally placed for stereoscopical purposes ; but it is not my present

DIAGRAM I.

purpose to do more than allude to stereoscopic modifications in passing.

The *Upright* is solidly and inseparably attached to the end of the Baseboard at right angles thereto, and constitutes the vertebral column of the apparatus. It is convenient to have a scale marked on it of inches or centimetres.

The *Brackets* move freely along the upright as shown, and can be clamped thereon at any point by screws; these screws are not shown, being on the far side. The *lower Bracket* has (at the central end vertically over the point X) a plummet on a string (P. L.). This plumb-line has nothing to do with the centreing of the plate and radiographic centre, but has to do with the centreing and adjustment of the patient along the principal radiographic axis O O′. When the patient lies, or any part of him, across the Baseboard properly orientated, then when the chosen bony point comes under the point of the plummet, we know that the patient or his limbs are as far as possible in identical positions to what we desire, or to what he previously occupied. When the Baseboard is used off the level, then instead of the plumb-line we would require to employ a pointed rod in a sleeve directed along OO′. The lower Bracket can be unshipped, and this must be done, of course, before the X-ray exposure is made. The *upper Bracket* is the essential one. It bears at its inner end a ring-shaped support for the focus tube, parallel to the plane of the Baseboard; the centre of the ring (n) must be accurately adjusted once for all vertically over the point X on the Baseboard. The focus tube is of the spherical bulb pattern now readily procurable, in which the radiographic centre of emission (R.C.) is accurately situated at the centre of this spherical portion. When so manufactured we know that however the tube may be tilted about when seated in the ring support, the radiographic centre must keep its position in O O′ always opposite the centre of the Baseboard, X.

We have thus by structural arrangement of parts secured that R.C. and n and X are permanently in the same straight line O O′, though R.C. can be varied at will in its movements to and fro in regard to the Baseboard. To determine the distance of R.C. from X, we have only to measure directly the distance of the lowest point of the spherical bulb from X, and add thereto the probably known and in any case easily measureable half external diameter of the spherical bulb of the focus tube.

In cases where it is preferred to have the focus tube below the patient and the plate or screen above, some such arrangement as that indicated in Diagram II would be found convenient.

A ready means of determining accurately by one direct measurement the position of the radiographic centre to a known part of the X-ray picture plane must be generally useful. I will now briefly suggest a few special purposes for which it may be employed.

DIAGRAM II.

Firstly, for *Proportional Representation.*[1] If a part of the body twelve inches thick, as measured between conspicuous bony points in a person of large size, be X-rayed for comparison with the corresponding part in another individual ten inches thick, but externally similar, then we cannot be sure from observed dissimilarity of outlines upon the photograph that there is a real

[1] "Proportional Representation," *Archives of the Röntgen Ray*. October, 1910.

dissimilarity or disproportion of internal parts X-rayed, unless the exposures are made at proportional distances of the radiographic centre from the corresponding parts of the plates. If the distance of the radiographic centre (R.C.) from the Baseboard in the first case were eighteen inches, then we should not get a reliable *prima facie* comparison with the smaller person's

DIAGRAM III.

outlines (or *vice versa*, whichever is taken as the standard) unless the principal distance of the radiographic centre from the centre of the Baseboard were made as nearly as possible fifteen inches, and the same aspect of both patients being of course in contact with the plate or screen.

Secondly, equally important and essential is an exact reproduction of the original position of the radiographic centre by the use of a small luminous flame at a stationary point on the side of the picture plane, remote from but corresponding with the original position in regard to the plate or screen of the radiographic centre at the time of exposure. If the position of the small luminous flame can be thus accurately adjusted, then it will be possible by the use of a small mirror on the surface of the screen or print, or by highly glazing the surface of the print itself, to determine the proper direction to search for a foreign body. When, for instance, the reflection of the small luminous flame is found to fall over the centre of the shadow of the foreign body as seen by the observer, then the eye, the centre of the shadow of the foreign body, and the foreign body itself must all be in line with the original stationary point of the radiographic centre.

In Diagram III a highly-glazed print is actually employed for this method of "centroscopy." In addition are figured some simple apparatus including sights and a small circular plane mirror with a small central perforation, mounted on a holder and suitable for use either with the screen or print. The glass of the mirror ought, of course, to be lead glass.[1]

Lastly, we may usefully imitate for diagnostic purposes the X-ray shadows of an internal organ or a deeply-placed bone by means of visible shadows thrown by a small luminous flame of a model of the organ in question or of a dried bone externally. In this case also an exact knowledge of the original position of the radiographic centre to the plate and object at the time of taking will be found equally advantageous in making an accurate diagnosis.[2]

[1] "The Fluoroscopic Diagnosis of Direction by a Plane Mirror upon the Screen," *Practitioner*, 1911, lxxxvi. 725.

[2] "Radiographic Estimation of Simple Enlargement by Means of Visible Shadows," *Bristol M. Chir. J.*, 1910, xxviii. 226.

## Figures and Tables

**Diagram I. f1:**
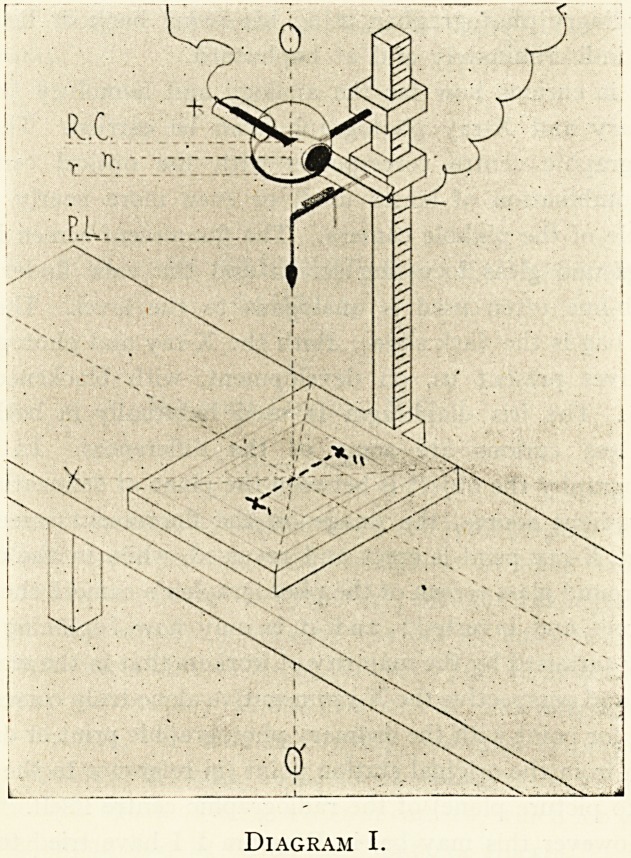


**Diagram II. f2:**
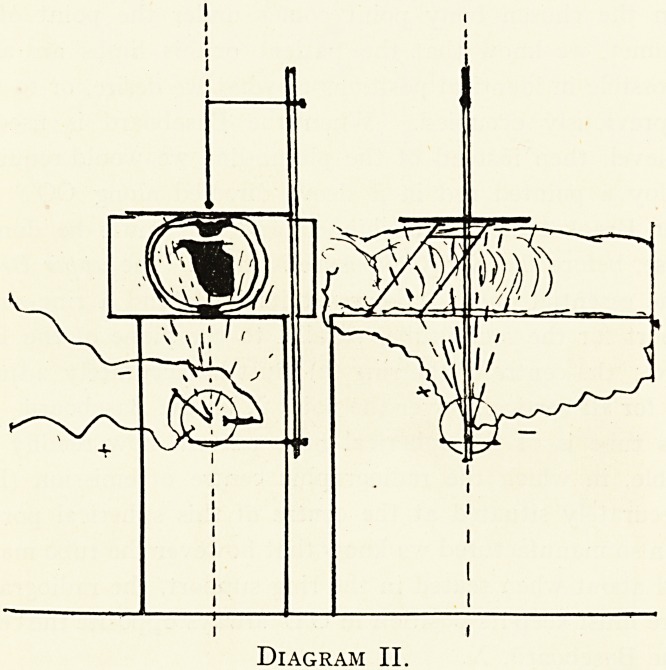


**Diagram III. f3:**